# Occupational and Environmental Carcinogenesis

**DOI:** 10.3390/cancers12092547

**Published:** 2020-09-08

**Authors:** Caterina Ledda, Venerando Rapisarda

**Affiliations:** Occupational Medicine, Department of Clinical and Experimental Medicine, University of Catania, Via Santa Sofia, 87–95123 Catania, Italy; venerando.rapisarda@unict.it or

**Keywords:** cancer, carcinogenesis, exposure, work, environment, risk, IARC

## 1. Occupational Issues

Occupational carcinogens have been shown to cause a considerable disease burden at a national and global level [1,2,3]. The WHO Comparative Risk Assessment project was the first attempt to produce comprehensive global estimates of the nature and extent of the burden of cancer arising from occupational exposures. In the year 2000, approximately 150,000 deaths were estimated due to past occupational exposure to 11 carcinogens (three cancer outcomes—lung, mesothelioma and leukemia) [4].

The identification of occupational carcinogens is significant for primary prevention, compensation and surveillance of exposed workers, in addition to recognizing causes of cancer in the general population [5]. International Agency for Research on Cancer (IARC) Monographs covering the years 1971–2020, use specific criteria to ensure occupational relevance and provide high confidence in the causality of observed exposure–disease associations [6]. A total of 120 agents were substances, mixtures or types of radiation classified in IARC Group 1 with ‘sufficient evidence of carcinogenicity’ in humans from studies of exposed workers and evidence of occupational exposure documented in the apposite monograph [6]. The number of recognized occupational carcinogens has augmented over time: these estimations are conventional and likely underestimate the quantity of carcinogenic agents present in workplaces. Exposure to these agents causes an extensive series of cancers (i.e., lung and other respiratory sites, skin). The main routes of exposure are inhalation and dermal contact. Significant progress has been made in identifying occupational carcinogens; however, there is a continuing need for research on the causes of work-related cancer. Most workplace exposures have not been evaluated for their carcinogenic potential due to inadequate epidemiologic evidence and a paucity of quantitative exposure data.

Moreover, 401 agents have been classified as “Probably carcinogenic to humans—Group 2A” or “Possibly carcinogenic to humans—Group 2B” [6]. On these agents it is essential that studies are made to deepen carcinogenesis and therefore be able to implement the right actions.

## 2. Environmental Issues

Risk of cancer can increase through exposure to cancer-causing agents, also referred to as “carcinogens”. These agents may be biological (specific viruses or bacteria), physical (ultraviolet light, X-rays) or chemical [7,8]. 

Moreover, lifestyle-related factors, screening and aging cannot fully account for the present overall growing incidence of cancer [7,8,9]. In order to propose the concept that in addition to lifestyle related factors, exogenous environmental factors may play a more important role in carcinogenesis than is expected, and may therefore account for the growing incidence of cancer, we overview herein environmental factors, rated as certainly or potentially carcinogenic by the International Agency for Research on Cancer (IARC) [6]. Chemicals related to environmental pollution appear to be of critical importance. Of major concern are: outdoor air pollution by carbon particles associated with polycyclic aromatic hydrocarbons; indoor air pollution by environmental tobacco smoke; formaldehyde and volatile organic compounds such as benzene and 1,3 butadiene, which may particularly affect children; and food pollution by food additives and by carcinogenic contaminants such as nitrates, pesticides, dioxins and other organochlorines. In addition, carcinogenic metals and metalloids, pharmaceutical medicines and cosmetics may be involved [7,8,9]. 

Involuntary exposure to carcinogens often comes to the public’s attention through reports in the media concerning particular issues (use of herbicides, contaminants of food, hazards associated with cosmetics etc.) However, in all such instances, the level of cancer risk is rarely made clear. Such reports can cause alarm and confusion, even though there may be no risk or minimal risk [7,8,9].

Although the risk fraction attributable to environmental factors is still unknown, this long list of carcinogenic and especially mutagenic factors supports our working hypothesis according to which numerous cancers may in fact be caused by the recent modification of our environment.

## 3. Future Directions

Carcinogenesis is a complex, multistep process, involving accumulation of genetic and epigenetic alterations that confer a growth and/or survival advantage, through which cells gradually achieve unchecked growth and eventually become fully malignant and invasive. There are numerous sources of physical, chemical, and biological exposures that stem from endogenous and exogenous sources—including occupational settings—that can induce such genetic and epigenetic alterations. This damage is repaired through a high-fidelity DNA repair process that operates through multiple pathways, although the system is imperfect and varies by repair mechanism, potentially resulting in incorporation of DNA damage and epigenetic alterations. The aim is clarifying the mechanisms of environmental and occupational carcinogenesis and DNA repair, and provides examples of physical and chemical carcinogens and epigenetic effectors.
